# Two-year neurocognitive responses to first occupational lead exposure

**DOI:** 10.5271/sjweh.3940

**Published:** 2021-03-31

**Authors:** Yu-Ling Yu, Lutgarde Thijs, Nelly Saenen, Jesus D Melgarejo, Dong-Mei Wei, Wen-Yi Yang, Cai-Guo Yu, Harry A Roels, Tim S Nawrot, Gladys E Maestre, Jan A Staessen, Zhen-Yu Zhang

**Affiliations:** Research Unit Hypertension and Cardiovascular Epidemiology, KU Leuven Department of Cardiovascular Sciences, University of Leuven, Leuven, Belgium.; Department of Cardiology, Guangdong Provincial Cardiovascular Institute, Guangdong Provincial People’s Hospital, Guangzhou, China; Centre for Environmental Sciences, Hasselt University, Diepenbeek, Belgium.; Department of Cardiology, Shanghai General Hospital, Shanghai Jiao Tong University School of Medicine, Shanghai, China.; Department of Endocrinology, Beijing Lu He Hospital and Key Laboratory of Diabetes Prevention and Research, Capital Medical University, Beijing, China.; Department of Neurosciences and Department of Human Genetics, University of Texas Rio Grande Valley School of Medicine, Brownsville, TX, USA.; Alzheimer´s Disease Resource Center for Minority Aging Research, University of Texas Rio Grande Valley, Brownsville, Texas, USA.; Research Institute Association for the Promotion of Preventive Medicine, Mechelen, Belgium.; Biomedical Science Group, Faculty of Medicine, University of Leuven, Leuven, Belgium

**Keywords:** digit-symbol test, neurocognitive function, occupational exposure, Stroop test

## Abstract

**Objectives:**

Lead exposure causes neurocognitive dysfunction in children, but its association with neurocognition in adults at current occupational exposure levels is uncertain mainly due to the lack of longitudinal studies. In the Study for Promotion of Health in Recycling Lead (NCT02243904), we assessed the two-year responses of neurocognitive function among workers without previous known occupational exposure newly hired at lead recycling plants.

**Methods:**

Workers completed the digit-symbol test (DST) and Stroop test (ST) at baseline and annual follow-up visits. Blood lead (BL) was measured by inductively coupled plasma mass spectrometry (detection limit 0.5 µg/dL). Statistical methods included multivariable-adjusted mixed models with participants modelled as random effect.

**Results:**

DST was administered to 260 participants (11.9% women; 46.9%/45.0% whites/Hispanics; mean age 29.4 years) and ST to 168 participants. Geometric means were 3.97 and 4.13 µg/dL for baseline BL, and 3.30 and 3.44 for the last-follow-up-to-baseline BL ratio in DST and ST cohorts, respectively. In partially adjusted models, a doubling of the BL ratio was associated with a 0.66% [95% confidence interval (CI) 0.03–1.30%; P=0.040] increase in latency time (DST) and a 0.35% (95% CI -1.63–1.63%; P=0.59) decrease in the inference effect (ST). In fully adjusted models, none of the associations of the changes in the DST and ST test results with the blood lead changes reached statistical significance (P≥0.12).

**Conclusions:**

An over 3-fold increase in blood lead over two years of occupational exposure was not associated with a relevant decline in cognitive performance.

Lead is a ubiquitous environmental toxicant. The Global Burden of Disease study assumed a causal association between intellectual disability and lead exposure in children (1), mainly justified by a participant-level meta-analysis involving 1333 children enrolled in seven population-based studies and followed up from birth or infancy until 5–10 years of age (2). The IQ point decrements associated with blood lead increments from 2.4–10, 10–20, and 20–30 μg/dL were 3.9, 1.9, and 1.1, respectively (2). The lead-associated intellectual decrement in children with a maximal blood lead level <7.5 μg/dL was greater than that observed in those with a maximal blood lead level of ≥7.5 µg/dL (P=0.015). These counterintuitive findings might be a product of residual confounding, falling exposure levels over time or a decreasing vulnerability for cognitive impairment with higher age (2). Turning to adults, the literature relating neurocognitive function to lead exposure in studies of the general population (3–9) or workers (10–13) with a cross-sectional (3–5, 7, 8, 10), case–control (11, 13) or longitudinal design (6, 9, 12) is contradictory. Similarly, two systematic reviews (14, 15), including 22 studies of exposed and unexposed workers but using different statistical methods, concluded that there was an inverse (14) or a null (15) association between neurocognition and occupational lead exposure. Unexposed and exposed blood lead levels in workers were unavailable in over ten studies (15). None of the studies compared blood lead levels before and after exposure (15). None of the individual studies was conclusive. Lack of true measures of the pre-occupational exposure and observer and publication bias were other issues obscuring the true relation between neurocognitive function and lead exposure for blood lead levels <70 µg/dL (15). Given the contradictory results of individual studies (3–13) and literature reviews (5, 14, 15), we identified a great need for prospective studies that would account for variability between people by comparing test results before and after lead exposure In the Study for Promotion of Health in Recycling Lead (SPHERL; NCT02243904) (16), we assessed the association between neurocognitive function and blood lead in young workers prior to (17) and up to two years after starting first occupational exposure.

## Methods

### Participants

SPHERL is a longitudinal study of newly hired lead workers at battery manufacturing and lead recycling plants in the United States (16). SPHERL complies with the Helsinki Declaration for investigations in humans (18). The Ethics Committee of the University Hospitals Leuven (Belgium) approved the study protocol. The health of the labor force was protected in compliance with the US Occupational Safety and Health Administration Standard (www.osha.gov/laws-regs/regulations/standardnumber/1910/1910.1025), which includes regular health check-ups, proper workplace ventilation, and the obligatory use of personal protective equipment. The two-year neurocognitive responses to first occupational lead exposure were a predefined secondary study endpoint (16).

**Figure 1:**
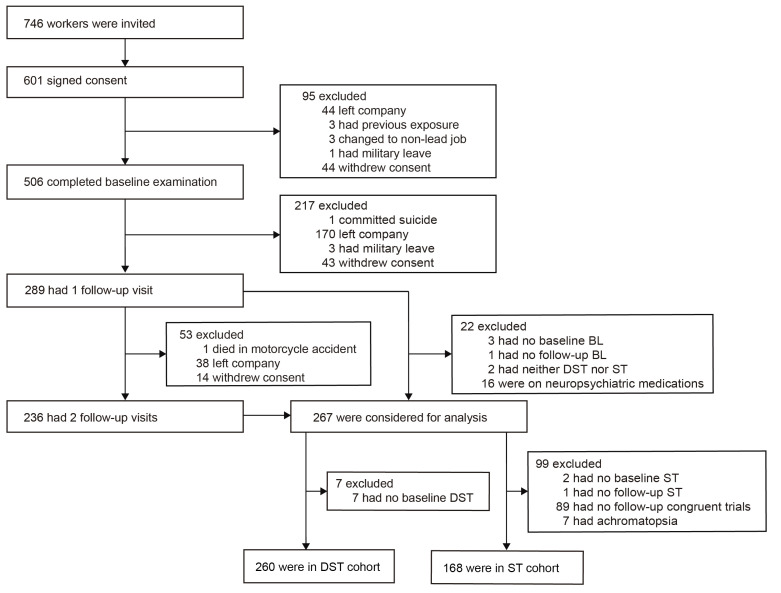
Flow chart. [BL=blood lead; DST=digit-symbol test; ST=Stroop test].

Of 746 newly hired workers invited to participate, 601 (80.6%) consented. However, in the interval between consent and the planned baseline examination (median, 19 days; 5–95^th^ percentile interval, 9–59 days), 95 laborers left the workplace or withdrew. From 25 January 2015 until 19 September 2017, 506 workers underwent the baseline examination, of whom 289 (57.1%) had one and 236 (46.6%) had two follow-up visits (figure 1). Of 289 participants with at least one follow-up visit, 22 were disqualified for analysis because blood lead had not been measured at baseline (N=3) or follow-up (N=1), because both the digit-symbol test (DST) and Stroop test (ST) had not been administered (N=2), or because workers were on neuropsychiatric medications (N=16), including antidepressants, amphetamines, sedatives, recreational drugs and/or opioids. Of the 267 analyzed participants, 7 were excluded from the DST cohort, because of missing baseline DST; 99 were excluded from the ST cohort, because of missing ST at baseline (N=2) or follow-up (N=1), missing congruent trials at follow-up (N=89), or because they had achromatopsia (N=7). The statistical analysis therefore included 260 participants in the DST cohort and 168 in the ST cohort with both a baseline and at least one follow-up assessment of their cognitive function and simultaneous blood lead measurements.

### Clinical measurements

At the study sites, trained nurses measured the workers’ anthropometric characteristics and applied current guidelines to measure office blood pressure at the brachial artery. After the workers had rested for five minutes in the sitting position, the nurses obtained five consecutive blood pressure readings to the nearest 2 mm Hg by auscultation of the Korotkoff sounds, using standard mercury sphygmomanometers. For analysis, the five readings were averaged. Blood pressure was categorized according to the 2017 ACC/AHA guideline (19). If systolic and diastolic blood pressure were in different categories, the highest value was used to classify participants. Heart rate was counted over 15 seconds. Body mass index (BMI) was body weight in kilograms divided by height in meter squared. The study nurses administered a validated (20) questionnaire at baseline and follow-up to collect information about each worker’s medical history, exposure to heavy metals, smoking and drinking habits, intake of medications, and educational attainment. Alcohol consumption was categorized as absent, light, moderate or heavy. The thresholds for the daily alcohol consumption self-reported by questionnaire were ≤6, >6–14 and >14 gram in women and ≤12, >12–28 and >28 gram in men.

### Neurocognitive function tests

The neurocognitive examination was conducted in a quiet air-conditioned room. We administered the computerized version of the DST and ST as published by Xavier Educational Software Ltd, Bangor, Wales, UK, using a laptop with touch screen. A video uploaded at the journal’s website illustrates the administration of these tests.

The DST measures processing speed, working memory, visuospatial processing, and attention (21). Participants performed the DST test at baseline and follow-up to assess the impact of lead exposure on general cognitive functions such as processing speed, working memory, visuospatial processing, and attention. A row of nine symbols paired vertically with nine digits was displayed at the top of the computer screen. The same symbols were also presented at the bottom of the screen but in a different order. The task is to touch as fast as possible the symbol at the bottom of the screen that is paired with the displayed digit. During the test, 36 digits appear one after one in the center of the screen. The worker performing the test has to provide the correct response before a new digit is presented. The time needed to complete the test, called latency, and the total number of errors served as measures of performance.

The ST was used to measure the impact of lead exposure on the Stroop effect, which is related to selective attention. Workers saw the printed name of a color and four buttons displayed in yellow, red, blue and green on the laptop screen. In congruent trials, the name of the color was printed in the matching color (eg, “yellow” was printed in yellow). In incongruent trials, the name of the color was printed in a different color (eg, “yellow” was printed in red). The task consists of touching the screen button with the color matching the printed color name as fast and accurately as possible, ignoring the color of the printed color name. The ST consisted of 4 congruent and 12 incongruent trials. Before the test, participants completed four practice trials. The mean reaction time (ms) is the average time that passed between the appearance of the color name and touching the correct button in congruent and incongruent trials, respectively. The main outcome measure in the Stroop test is the inference effect, calculated as the ratio of the mean reaction time for the incongruent to the congruent trials, which is equivalent to the antilog of the difference between the log transformed reaction times. The inference score is defined as the proportion of the correct answers in congruent trials minus the proportion of correct answers in incongruent trials.

### Biochemical measurements

Venous blood samples were obtained after 8–12 hours of fasting. Blood lead levels were determined on whole blood by inductively coupled plasma mass spectrometry at an analytical laboratory certified for blood lead analysis in compliance with the provisions of the OSHA Lead Standard, 29CFR 1910.1025 (Occupational Safety and Health Administration [www.osha.gov]). Prior to analysis, the specimens were digested by nitric acid and spiked with an iridium internal standard. The detection limit was 0.5 µg/dL. The accuracy of the lead tests was verified by use of proficiency samples purchased from the College of American Pathologists (CAP) and the Pennsylvania Department of Blood Lead Programs (22). Proficiency testing was performed in six separate trial runs, including in total 30 test samples annually. All survey materials were handled in the same manner as the study samples and processed with the normal workflow, utilizing the same repeat/dilution protocols and calibration and quality control frequency (22). Compliance with the Clinical Laboratory Improvement Amendments (CLIA), CAP and the New York State accreditation and regulatory requirements was verified routinely with test level review of the laboratory services by external auditors. Calibrators with certified accuracy (National Institute of Standards and Technology [www.nist.gov]) were included in each batch of study samples and spanned the range of the analytical measurement range. Accuracy was evaluated by Westgard Rules (23) and defined within the total allowable error established with review of the CAP, Centers for Disease Control and Prevention, CLIA 88 (24), and OSHA guidelines. Accuracy, defined as the deviation from known lead standards ran along with the study samples, was within 10% (22). The bias determined according to the Bland & Altman approach (25) in 30 spilt blood samples with blood lead concentrations (average in duplicate samples) ranging from 0.70–27.9 µg/dL, was 0.08 µg/dL [95% confidence interval (CI) -0.01–0.18, P=0.078; supplementary material www.sjweh.fi/show_abstract.php?abstract_id=3940, figure S1) (17). The repeatability coefficient, defined as twice the SD of the signed differences between duplicate measurements (25), was 0.52. Expressed as a percentage of the mean blood lead concentration or as a percentage of near maximal variation in blood lead (four times the SD of the logarithmically transformed distribution), the repeatability coefficients were 6.7% and 1.9%, respectively. Lower values indicate better repeatability.

Serum total and high-density lipoprotein (HDL) cholesterol, serum creatinine, and blood glucose were measured by automated enzymatic methods and serum insulin by ELISA. Over three evaluations, the laboratory obtained a proficiency score of 100% for blood lead and 100% for routine biochemistry. Diabetes mellitus was a self-reported diagnosis, a fasting blood glucose of 126 mg/dL (7.0 mmol/L) or higher, or use of antidiabetic drugs.

### Statistical analysis

For database management and statistical analysis, we used the SAS software, version 9.4, maintenance level 5 (SAS Institute Inc, Cary, NC, USA). Departure from normality was evaluated by the Shapiro-Wilk statistic. Skewness and kurtosis were computed as the third and fourth moments about the mean divided by the cube of the standard deviation. We applied a logarithmic transformation (base 10) to normalize the distributions of latency time (DST), mean reaction time and interference effect (ST), and blood lead. We reported the central tendency and spread of continuously distributed variables as mean with standard deviation (SD) or for logarithmically transformed variables as geometric mean with interquartile range (IR) or with the 5–95^th^ percentile interval. To compare means and proportions, we applied the t-statistic or ANOVA for continuous variables, and the Fisher exact test for categorical variables, respectively.

In exploratory analyses, we assessed the results of DST and ST across fourths of the follow-up-to-baseline blood lead concentration ratio. Changes in DST and ST were correlated with the corresponding changes in log blood lead using a random intercept mixed model, accounting for the correlations between repeated observations within the same participant. A compound symmetry correlation structure was assumed and variance parameters were estimated using restricted maximum likelihood. The model included change in log blood lead as a fixed effect. Neurocognitive responses to the changes in blood lead were expressed for a doubling of the follow-up-to-baseline blood lead concentration ratio. For each outcome, unadjusted, partially and fully adjusted models were constructed. Partially adjusted models included sex, age and the neurocognitive function test at baseline as covariables. Fully adjusted models additionally accounted for ethnicity (white versus other), change in age, baseline BMI, changes in body weight, educational attainment, baseline blood lead and the baseline values and changes during follow-up in smoking status, and the total-to-HDL serum cholesterol, and alcohol consumption (light, moderate and heavy drinkers). Covariables were selected on the basis of their associations with both neurocognitive function and blood lead in previous publications (26–29). In sensitivity analyses, we stratified the study participants according to median age, the median baseline blood lead level and the median cumulative blood lead index (CBLI) (30). We also checked the performance of the mixed models by relating changes in neurocognitive function and blood lead separately for the 1- and 2-year visits by means of linear regression.

## Results

### Characteristics of participants

Of 260 participants, 229 (88.1%) were men, 122 (46.9%) were white, 117 (45.0%) were Hispanic, and 21 (8.1%) had other self-reported ethnicities. At baseline, age averaged 29.4 years (supplementary figure S2), BMI 28.8 kg/m^2^, serum creatinine 0.93 mg/dL, total and HDL serum cholesterol 171.8 mg/dL and 46.8 mg/dL, the total-to-HDL cholesterol ratio 3.90, and blood glucose 93.8 mg/dL (supplementary table S1). The cohort included 6 women and 63 men, who were current smokers (N=69; 26.5%); 11 women and 102 men (N=113; 43.4%) reported alcohol intake, of whom 9 and 65, 2 and 23, 0 and 14 were light, moderate and heavy drinkers, respectively. The baseline characteristics of the 168 workers in the ST cohort were similar (supplementary table S2). The characteristics of 267 workers included in the DST or ST cohort or both and the 239 workers not analyzed were largely similar (supplementary table S3).

### Blood lead

**Figure 2:**
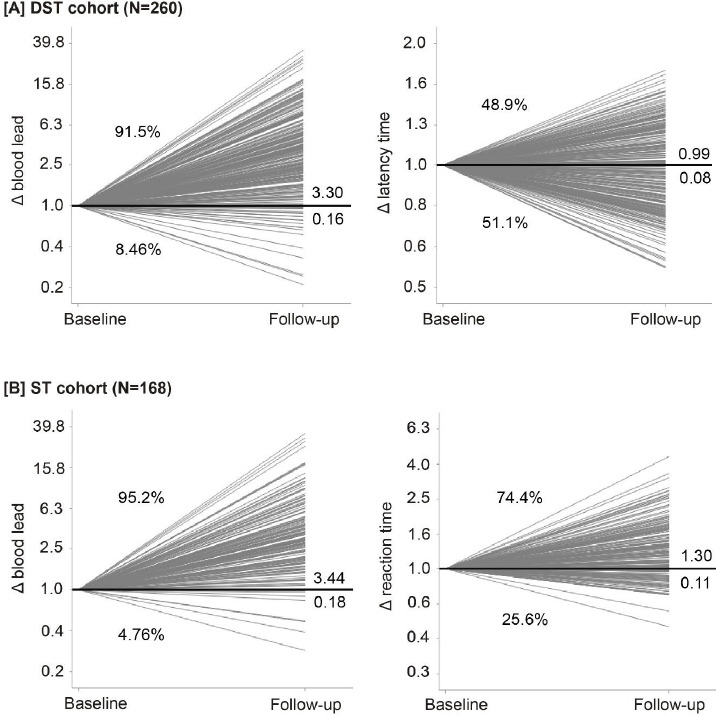
Baseline-to-last-follow-up ratios (Δ) in blood lead [A, B], latency time in the digit-symbol test in DST cohort [A], and mean reaction time in the incongruent trials in ST cohort [B]. [DST=digit-symbol test; ST=Stroop test]. Numbers at the right side of the line graphs represent the mean ratio (above the unity line) and its SE (below the unity line). Percentage values represent the number of workers with a ratio greater than or less than unity.

Median follow-up was 2.0 [5–95^th^ percentage interval (PI) 1.0–2.2] years. In the DST cohort, the geometric mean blood lead concentration was 3.97 (PI 0.90–14.3) μg/dL at baseline, 13.4 (PI 3.70–30.3) μg/dL and 12.8 (PI 2.80–29.2) μg/dL at the first and second follow-up visits, respectively. The corresponding blood lead levels in the ST cohort were 4.13 (PI 1.20–13.0) μg/dL, 14.4 (PI 4.60–30.3) μg/dL and 16.1 (PI 5.40–31.5) μg/dL. The last-follow-up-to-baseline blood lead concentration ratio averaged 3.30 (PI 0.79–14.9) and 3.44 (PI 1.01–13.8) in DST and ST cohorts, respectively (figure 2 and supplementary figure S4). The increase in the blood lead concentration was fully observed at the 1-year follow-up visit (supplementary figure S3).

### Digit-symbol test

Workers with a completely correct DST numbered 153 (58.9%) at baseline and 160 (61.5%) at the last follow-up examinations. Among all participants, the geometric mean test duration was 108.9 [interquartile range (IQR) 95.8–120.8] seconds at baseline and 107.6 (IQR 91.4–122.6) seconds at last follow-up. The number of errors and the mean latency time were similar at baseline and last follow-up (table 1). Across fourths of the distribution of blood lead changes, trends in baseline (P≥0.076; supplementary table S4), follow-up (P≥0.38; supplementary table S5) and the longitudinal changes (P≥0.079; table 2) of errors and of the mean latency time were not significant. In mixed models (supplementary figure S5) only accounting for clustering within participants (P=0.0033; table 3) and in partially adjusted models (P=0.040) also adjusted for sex, age and the baseline test result, the change in latency time increased with the follow-up-to-baseline blood lead concentration ratio. However, in a fully adjusted model, this association size weakened to 0.55% (95% CI -0.33–1.42; P=0.22; table 3 and supplementary figure S5). Moreover, in unadjusted, partially adjusted and fully adjusted model, none of the odd ratios for an increasing error rate reached significance (P≥0.12; table 3).

**Table 1:** Baseline and follow-up neurocognitive tests in the digit-symbol test (DST) and Stroop test (ST) cohorts. Average values are geometric means [interquartile range (IQR)]. [CI=95% confidence interval; MRT=mean reaction time].

Characteristic	Baseline		Follow-up		∆ (95% CI) ^a^		P-value
		
	N (%)	Mean (IQR)	N (%)	Mean (IQR)	Mean	95% CI	

DST cohort (N=260)							
Mean latency time (s, log)		108.9 (95.8–120.8)		107.6 (91.4–122.6)	-1.17	4.11–1.86	0.44
Number of errors							
0	153 (58.9)		160 (61.5)		2.69	–5.27–10.6	0.74
1	73 (28.1)		71 (27.3)		-0.77	–8.68–7.15	
>1	34 (13.1)		29 (11.2)		-1.92	–7.25–3.43	
ST cohort (N=168)							
MRT in incongruent trials (ms, log)							
All responses		1606 (1309–1917)		2088 (1666–2525)	30.1	22.7–37.9	<0.0001
Correct responses ^b^		1608 (1307–1922)		2077 (1636–2535)	29.8	22.3–37.8	<0.0001
MRT in congruent trials (ms, log)							
All responses		1485 (1181–1714)		1979 (1563–2458)	33.3	24.4–42.8	<0.0001
Correct responses ^b^		1485 (1181–1714)		1990 (1574–2476)	34.0	25.0–43.6	<0.0001
Correct ratio in incongruent trials (%)							
100	145 (86.3)		138 (82.1)		-4.17	–11.8–3.59	0.55
90–99	12 (7.14)		17 (10.1)		2.98	–3.25–9.13	
<90	11 (6.55)		13 (7.74)		1.19	–3.98–6.33	
Correct ratio in congruent trials (%)							
100	168 (100.0)		164 (97.6)		-2.38	–5.15–0.45	0.044
<100	0 (0.0)		4 (2.38)		2.38	–0.45–5.15	
Interference effect (log)							
All responses		1.08 (0.96–1.21)		1.06 (0.90–1.22)	-2.40	–7.36–2.82	0.36
Correct responses ^b^		1.08 (0.97–1.21)		1.05 (0.87–1.22)	-3.08	–8.24–2.37	0.26
Interference score							
<0	0 (0.00)		3 (1.79)		1.79	–0.80–4.33	0.14
0	145 (86.3)		136 (80.9)		-5.36	–13.2–2.57	
>0	23 (13.7)		29 (17.3)		3.57	–4.10–11.2	

a Changes from baseline to last follow-up were given with 95% CI. For proportions, categorical variables and logarthmically transformed variables, percentage changes are given.

b One participant did not provide any correct response at baseline and follow-up and was not included in the MRT of correct responses.

**Table 2:** Changes (Δ) from baseline to follow-up in the neurocognitive responses by fourths of the distribution of follow-up-to-baseline blood lead concentration ratio. [PI=5–95^th^ percentile interval; DST=digit-symbol test; ST=Stroop test; MRT=mean reaction time].

Characteristic ^a^	Low fourth		Low-middle fourth		High-middle fourth		High fourth		P for linear trend
			
	Mean/ Median	PI	Mean/ Median	PI	Mean/ Median	PI	Mean/ Median	PI	
DST cohort (N=260)								
Quartile limits		<1.90		1.90-3.37		3.37-5.75		>5.75	
∆ latency time (%)	-7.06	-41.7–43.8	1.59	-30.3–51.6	-0.28	-33.9–42.5	1.33	-27.6–38.7	0.079
∆ number of errors	0.0	-2.0–1.0	0.0	-3.0–2.0	0.0	-1.0–2.0	0.0	-1.0–1.0	0.13
ST cohort (N=168)									
Quartile limits		<1.98		1.98-3.26		3.26-5.52		>5.52	
∆ MRT in incongruent trials									
All responses (%)	39.9	-9.32–169	35.0	-25.7–162	33.7	-25.8–102	13.4	-28.2–160	0.015
Correct responses (%) ^b^	42.4	-6.09–175	34.6	-25.7–162	35.3	-25.8–102	9.32	-31.5–159	0.0037
∆ MRT in congruent trials									
All responses (%)	49.6	-19.8–231	35.5	-23.7–158	23.7	-33.7–128	25.8	-34.6–175	0.051
Correct responses (%) ^b^	49.6	-19.8–231	36.9	-23.7–158	23.7	-33.7–128	27.3	-34.6–175	0.061
∆ number of errors in incongruent trials	0.0	-1.0–1.0	0.0	-2.0–5.0	0.0	-1.0–2.0	0.0	-1.0–1.0	0.34
∆ number of errors in congruent trials	0.0	-0.0–0.0	0.0	-0.0–0.0	0.0	-0.0–0.0	0.0	-0.0–0.0	0.37
∆ interference effect									
All responses (%)	-6.53	-37.1–37.9	-0.38	-41.5–84.6	8.12	-32.6–71.2	-9.88	-46.1–70.7	0.91
Correct responses (%) ^b^	-5.80	-36.8–45.1	-2.40	-43.8–79.5	9.42	-31.3–90.4	-12.8	-51.8–83.1	0.65

a Values are geometric means (reported as percent change) and PI for logarithmically transformed variables, and median and PI for ordinal variables.

b One participant did not provide any correct response at baseline and follow-up and was not included in the MRT of correct responses.

**Table 3:** Associations between changes (∆) from baseline to follow-up in neurocognitive function and in blood lead. [OR=odds ratio; CI=confidence interval; DST=digit-symbol test; ST=Stroop test].

Characteristic	Unadjusted				Adjusted ^a^				Fully adjusted^b^			
		
	% ^c^	OR ^c^	95% CI	P-value	% ^c^	OR ^c^	95% CI	P-value	% ^c^	OR ^c^	95% CI	P-value
DST cohort (N=260)												
∆ latency time (%)	1.17		0.39–1.95	0.0033	0.66		0.03–1.30	0.040	0.55		-0.33–1.42	0.22
Increasing error rate (0,1)		1.04	0.87–1.25	0.65		1.00	0.83–1.21	0.96		1.28	0.94–1.76	0.12
ST cohort (N=168)												
∆ MRT in incongruent trials												
All responses (%)	-2.03		-3.91–-0.11	0.039	-1.95		-3.48–-0.39	0.016	-0.83		-3.20–1.59	0.49
Correct responses (%) ^d^	-2.65		-4.55–-0.70	0.0092	-2.23		-3.76–-0.68	0.0061	-1.26		-3.59–1.13	0.29
∆ MRT in congruent trials												
All responses (%)	-1.57		-3.85–0.76	0.18	-1.64		-3.41–0.15	0.072	-1.56		-4.32–1.28	0.27
Correct responses (%) ^d^	-1.55		-3.83–0.79	0.19	-1.61		-3.37–0.18	0.077	-1.54		-4.29–1.30	0.28
Increasing error rate ^e^												
Incongruent trials (0,1)		0.76	0.54–1.07	0.11		0.72	0.50–1.04	0.078				
Congruent trials (0,1)		1.25	0.55–2.87	0.59								
∆ Interference effect												
All responses (%)	-0.43		-2.16–1.33	0.62	-0.35		-1.63–0.94	0.59	1.08		-0.97–3.17	0.29
Correct responses (%)	-0.71		-2.52–1.14	0.44	-0.45		-1.76–0.87	0.49	1.05		-1.03–3.17	0.32

a Adjusted models accounted for sex and baseline age and the baseline neurocognitive test results, ie, latency/reaction time (continuous outcomes) or the number of errors (ordinal outcomes).

b Fully adjusted models additionally accounted for ethnicity (white vs other), change in age, baseline body mass index, change in body weight, educational attainment, baseline blood lead, and the baseline values of and changes during follow-up in smoking status, alcohol intake (light, moderate and heavy), and the total-toHDL serum cholesterol ratio.

c All association sizes were expressed for a doubling of the baseline-to-follow-up blood lead concentration ratio. Estimates are the percentage difference in the follow- up minus the baseline value for continuous variables and odds ratios for categorical outcomes. Estimates were derived from mixed models, including both the 1-year and 2-year changes in neurocognitive function and blood lead, while accounting for within-subject correlations using a random participant effect.

d One participant did not provide any correct response at baseline and follow-up and was not included in the MRT of correct responses.

e An ellipsis indicates that the model did not converge.

We ran stratified analyses using fully adjusted models to evaluate the consistency of the changes of neurocognitive function among workers aged <26.4 and ≥26.4 years (supplementary table S6), baseline blood lead <4.20 and ≥4.20 μg/dL (supplementary table S7), and CBLI <32.5 and ≥32.5 μg/dL × year (supplementary table S8), respectively. In these stratified analyses, an increasing error rate in the high baseline blood lead subgroup was the only measurement, which tended to be associated with the follow-up-to-baseline blood lead concentration ratio: odds ratio, 1.68 (95% CI 0.99–2.86; P=0.056 in the high baseline blood lead stratum vs 1.00 in the low exposure group (95% CI 0.64–1.58; P=0.99) with a nonsignificant interaction (P=0.34; supplementary table S7). The results of the linear regression analyses correlating changes in latency time and blood lead separately at the 1- and 2-year follow-up visits largely confirmed the findings obtained by mixed models (supplementary table S9).

### Stroop test

The Stroop test with incongruent trials was completed error free in 145 (86.3%) workers at baseline, with no difference between baseline and follow-up in these proportions (P=0.55; table 1). The mean reaction time for incongruent trials increased from 1606 ms at baseline to 2088 ms at the last follow-up visit in all participants and from 1608 ms to 2077 ms, if only the correct responses were considered (P<0.0001). The changes from baseline to follow-up averaged 30.1% (CI 22.7–37.9; P<0.0001) and 29.8% (CI 22.3–37.8; P<0.0001), respectively (table 1). Supplementary tables S11 and S12 show the mean reaction time and blood lead levels at baseline and follow-up, and overall, in the workers tested and broken down by the attending observer. Supplementary table S11 illustrates the effect of the observer on test performance and supplementary table S12 reflects the unpaired distribution of observers between the baseline and follow-up examinations.

Across fourths of the distribution of the blood lead changes, there was a trend towards smaller increases in mean reaction time with larger increases in blood lead (P≤0.015; table 2). For congruent trials, 168 (100%) were completed with fully correct answers at baseline and 164 (97.6%) at follow-up visit with an increasing mean reaction time from 1485 ms to 1979 ms (P<0.0001, table 1). The geometric means of the interference effects were 1.08 at baseline and 1.06 at the last follow-up visit in all workers. The distributions of inference score were similar at baseline and last follow-up visits (P=0.14; table 1). For congruent trials, in unadjusted and in partially and fully adjusted models, irrespective of whether all trials or only the error-free trials were analyzed, there was no association between the changes in mean reaction time and those in blood lead (P≥0.072; table 3). For incongruent trails, in the unadjusted models only accounting for clustering within participants and in the partially adjusted models, the longitudinal change in mean reaction time decreased as the blood lead increasing. However, in the fully adjusted models, the association sizes for a doubling of blood lead were -0.83% (95% CI -3.20–1.59; P=0.49) in all trials and -1.26% (95% CI -3.59–1.13; P=0.29) in error-free trials, respectively (table 3 and supplementary figure S5). Moreover, there was no association between the changes in interference effect and those in blood lead (P≥0.29; table 3). In the analyses stratified by median age (27.0 years; supplementary table S5), median baseline blood lead (4.30 μg/dL; supplementary table S6), or median CBLI (33.3 μg/dL × year; supplementary table S7), associations were all nonsignificant (P≥0.096; interaction P≥0.22). Linear regression analysis of the 1-year data was confirmatory (supplementary table S9).

### Consistency between baseline and last follow-up data

Supplementary table S10 lists the associations between blood lead level and the performance of participants in the neurocognitive tests at baseline and the last follow-up separately. None of the association sizes (slopes) in unadjusted or adjusted analyses reached significance (P≥0.14), with no differences between baseline and last follow-up in the association sizes (P slope≥0.23).

## Discussion

In a real-world experiment, among workers without known previous occupational exposure and taking up new jobs in lead recycling and battery manufacturing plants in the United States, an over threefold increase in the blood lead concentration over the 2-year follow-up was not associated with worsening of cognitive function, as assessed by the DST and ST. These longitudinal findings are in keeping with the cross-sectional analysis of the baseline SPHERL data (supplementary table S10), which did not show any association between cognitive performance as assessed by the same tests and blood lead prior to occupational exposure (17). The longitudinal changes in mean reaction time in incongruent ST trials tended to correlate inversely with the corresponding changes in blood lead, similar to congruent trials (tables 2 and 3). To what extent training effects (31) or the interaction between observers and test takers (supplementary table S11) influenced the ST test performance cannot be ascertained. To exclude an effect of the cumulative lead dose, we ran analyses stratified by the medians of age, baseline blood lead or CBLI in both cohorts, which confirmed the main analysis.

Lead is a cumulative toxicant, which is for 90–95% stored in bone, from where it is recirculated with a half-life of 20–25 years (32, 33). Blood lead, for 95% carried by red blood cells, reflects recent exposure over the past 1–2 months and the amount of lead released and recirculated from bone (32). Bone lead correlates with blood lead (33, 34) and explains around 20% of the variance in blood lead, depending on seasonality (33) and hormonal and other endogenous and environmental stimuli, influencing the balance between bone formation and resorption (34). Recirculation of lead from bone explains why there is a lag time when occupational (32) or environmental (35) lead exposure drops. These lead toxicokinetics are important in the interpretation of our current results. A narrative review on the association of neurocognitive function and lead exposure compiled evidence from 21 studies published from 1996–2006. All studies had assessed bone and blood lead as biomarkers of internal exposure, 15 in occupational studies and 6 in environmental settings (5). At exposure levels representative of contemporary environmental exposure, associations of cognitive function with biomarkers of cumulative dose (mainly lead in tibia) were stronger and more consistent than associations with blood lead levels as assessed by concurrent, cumulative or peak blood lead levels (36). Conversely, studies of currently exposed workers generally found associations that were more apparent with blood lead levels (36). Given the persistence of lead in the human body, both bone and blood lead increase with advancing age (33, 34). Consequently, with advancing age, the blood lead concentration reflects environmental exposure levels stretching further back in time. In the United States (scienceprogress.org/2008/10/a-brief-history-of-lead-regulation), lead-containing paint was only effectively banned in 1976 and leaded gasoline was completely phased out only in 1995 (37). Mean blood lead levels in the United States decreased from 13.1 μg/dL in the National Health and Nutrition Examination Survey II (NHANES II; 1976–1980) (38) to 2.76 μg/dL in NHANES III (1988–1994) (39) and to 1.64 μg/dL in NHANES IV (1999–2002) (28). Our study moves the field forward because the cumulative lead dose in our young participants must reflect present-day environmental exposure levels and, as suggested in a systematic literature review (15), we addressed variability between people by comparing neurocognitive test results before and after occupational exposure.

Neurocognitive functions are integrated cognitive processes linked to multiple particular cerebral areas, neural pathways or cortical networks in the brain (40, 41). In this study, we evaluated the neurocognitive function, using two complementary tests, which are sensitive to detect mild cognitive impairment under lead exposure (31, 42). On the one hand, the DST assesses complex attention, motor speed, visual-perceptual functions and executive function (42). Functional magnetic resonance imaging (fMRI) studies in young healthy adults (43) and octogenarians (44) showed that taking the DST activated the frontal parietal cortical network, probably reflecting visual search and working memory processes (43, 44). The ST provides information on processing speed, selective attention, automaticity and parallel distributed processing (45–47). In fMRI studies, taking the ST activated the anterior cingulate, insula, premotor and inferior frontal brain regions (48).

### Strengths and limitations

The strong points of our study are its longitudinal design (15), the young age of its participants the starting blood lead level representative of current environmental exposure levels, and the stringent quality control of the blood lead concentration. However, our study also has limitations. First, the attrition rate among the 506 workers who participated in the baseline examination, but defaulted from follow-up amounted to 217 (42.9%). However, according to the SPHERL protocol (16), the anticipated attrition rate was estimated to be 50% and >500 workers had to be enrolled. We met these numbers. The baseline characteristics of workers included or not included in the analyses were to a large extent similar (supplementary table S3), so that it is unlikely that attrition significantly biased the study results, although bias due to unmeasured confounders can never be excluded. Second, the study was primarily powered for blood pressure and renal outcomes, while neurocognitive function was among the secondary outcomes. However, the association sizes between the changes in the neurocognitive indexes and blood lead were small and sample size does impact on significance levels, but has no direct link with estimates of association size. Third, due to a software error, the ST with congruent trails was only administered to 168 (62.9%) participants at follow-up. However, the results of the congruent and incongruent tests were consistent. Fourth, the observer-participant pairing was not standardized throughout the study (supplementary tables S11 and table S12), which might have introduced bias in the observed baseline to follow-up changes in neurocognitive function. Fifth, the median 2-year follow-up might have been too short for neurocognitive effects associated with lead exposure to become evident. For this reason, as anticipated (16) the cohort will be kept in follow-up for an additional two years. Finally, although the ethnic distribution of the workers was representative for the population at the recruitment sites, women were under-represented. Only 11.6% of 267 analyzed participants were female, which precluded analyses stratified by sex.

### Concluding remarks

At the exposure level in our study, we failed to demonstrate a consistent and significant association of changes in neurocognitive function in the workers with an over threefold increasing blood lead concentration over the 2-year follow-up. Lead exposure represents an occupational and environmental health hazard that should be addressed worldwide. Our findings and the contradictory literature (15) suggest that in adults there is no causal link between neurocognitive impairment and low-level lead exposure as reflected by blood lead levels below 30 µg/dL. In weight-of-the-evidence approaches, policy makers might account for our findings in setting thresholds for occupational and environmental lead exposure levels, so that the prevention resources of neurocognitive function are dedicated to the more important drivers of cognitive impairment, in particular low educational attainment, socio-economic deprivation, abuse of alcohol and recreational drugs, discriminating based on ethnicity, and not providing opportunities for immigrants to assimilate the skills necessary for social integration (29).

### Funding

The International Lead Association (www.ila-lead.org) provided an unrestricted grant to the Research Unit Hypertension and Cardiovascular Epidemiology, KU Leuven Department of Cardiovascular Sciences, University of Leuven, partially supporting database management and statistical analysis. The non-profit research institute Alliance for the Promotion of Preventive Medicine (www.appremed.org) received a grant from OMRON Healthcare Co. Ltd., Kyoto, Japan. The funding source had no role in the study design; in the collection, analysis, and interpretation of the data; or in the writing of the report. The corresponding author had full access to all data and had the final responsibility for the decision to submit for publication.

## Supplementary material

Supplementary video

Supplementary material
