# Supplementary material for: Two-year neurocognitive responses to first occupational lead exposure
Source: Scand J Work Environ Health. 2021 Mar 31;47(3):233–43. doi: 10.5271/sjweh.3940 (PMC8126443; doi:10.5271/sjweh.3940)
Supplement: Supplementary material [file SJWEH-47-233-S002.pdf]

Two-year neurocognitive responses to first occupational lead exposure[1]

by Yu-Ling Yu, MD, Lutgarde Thijs, MSc, Nelly Saenen, PhD, Jesus D Melgarejo, MD, Dong-Mei Wei, MD, Wen-Yi Yang, MD, PhD, Cai-Guo Yu, MD, Harry A Roels, PhD, Tim S Nawrot, PhD, Gladys E Maestre, MD, PhD, Jan A Staessen, MD, PhD,[2] Zhen-Yu Zhang, MD, PhD

1. *Supplementary material*
2. *Correspondence: to Jan A. Staessen, Research Institute Alliance for the Promotion of Preventive Medicine, Leopoldstraat 59, BE-2800 Mechelen, Belgium. [E-mail: jan.staessen@appremed.org or jan.staessen@med.kuleuven.be]*

Table of contents **Page**

**Cumulative blood lead index**		2
**Table S1.**	Baseline and follow-up characteristics of workers in the DST cohort	3
**Table S2.**	Baseline and follow-up characteristics of workers in the ST cohort	5
**Table S3.**	Baseline characteristics of workers analyzed and not analyzed	6
**Table S4.**	Baseline neurocognitive function by fourths of the distribution of the follow-up-to-baseline blood lead concentration ratio	9
**Table S5.**	Follow-up neurocognitive function by fourths of the distribution of the follow-up-to-baseline blood lead concentration ratio	11
**Table S6.**	Associations between changes from baseline to follow-up in neurocognitive function and blood lead stratified by the median baseline age	13
**Table S7.**	Associations between changes from baseline to follow-up in neurocognitive function and blood lead stratified by the median baseline blood lead concentration	14
**Table S8.**	Associations between changes from baseline to follow-up in neurocognitive function and blood lead stratified by the median CBLI	15
**Table S9.**	Associations between changes in neurocognitive function and blood lead from baseline to follow-up modelled by linear regression analysis.	16
**Table S10.**	Association of neurocognitive function with blood lead at baseline and last follow-up analysed separately, using linear of logistic regression.	17
**Table S11.**	Time-dependent DST and ST results by observer	18
**Table S11.**	Geometric mean blood lead levels in the DST and ST cohorts by observer	20
**Figure S1.**	Bland-Altman plot for duplicate blood lead measurements in 30 workers	21
**Figure S2.**	Baseline age distribution in the DST and ST cohorts	22
**Figure S3.**	Blood lead levels at baseline and at the 1-year and 2-year follow-up visits	23
**Figure S4.**	Distributions of baseline blood lead, follow-up blood lead, and the baseline-to-last-follow-up ratios in blood lead in the DST and ST cohorts	24
**Figure S5.**	Associations of the changes from baseline to last follow-up in latency time in the DST cohort and in mean reaction time in ST cohort with the follow-up-to-baseline blood lead concentration ratio	25

Cumulative blood lead index

The calculation of cumulative blood lead index (CBLI) is based on the blood lead level in early years of life and the repeated measurement before and after exposure, and the time intervals between successive blood lead measurements, which indicates the accumulative level of lead exposure. The formula for CBLI (μg/dL× year) is as follows (1).

$$\int_0^T Pb\_B_t\,dt = \sum 0.5(Pb\_B_i + Pb\_B_{i+1})\Delta t = \text{CBLI}$$

where Pb_B_i and Pb_B_{i+1} are the ith and $(i+1)^{th}$ blood lead levels, and Δt (in years) is the time interval between successive blood lead values (1). In our study, age for workers leaving school at less than, at, and above the 12th grade were assumed to be 14, 18 and 23 years; based on NHANES data (1988-1994), the corresponding blood lead levels in early years were set at 2.2, 1.4 and 1.5 μg/dL, respectively (2). Therefore, the CBLI in our study was:

$$\text{CBLI} = 0.5 \times (A_0 - A_e) \times (Pb_0 + Pb_e) + 0.5 \times (A_1 - A_0) \times (Pb_1 + Pb_0) + 0.5 \times (A_2 - A_1) \times (Pb_2 + Pb_1)$$

The symbols, e, 0, 1 and 2 in the equation above, represented the age (A) and blood lead level (Pb) early in life, at baseline, and at the 1-year and 2-year follow-up visits, respectively. If the participant missed one follow-up visit, the last term of the above equation was dropped.

References

1. Roels H, Konings J, Green S, Bradley D, Chettle D, Lauwerys R. Time-integrated blood lead concentration is a valid surrugate for estimating the cumulative lead dose assessed by tibial lead measurement. Environ Res. 1995;69:75-82.

2. Fadrowski JJ, Navas-Acien A, Tellez-Plaza M, Guallar E, Weaver VM, Furth SL. Blood lead level and kidney function in US adolescents. The Third National Health ans Nutrition Examination Survey. Arch Intern Med. 2010;170:75-82.

Table S1. Baseline and follow-up characteristics of workers in the DST cohort (starts). Averge values are arithmetic [standard deviation (SD)] or geometric means [interquartile range (IR)]. [CI=confidence interval; HDL=high density lipoprotein; DST=digit-symbol test].

Characteristic (N=260)	Baseline		Last follow-up		△ (95% CI)[a]		P value
	N (%)	Mean (SD/IR)	N (%)	Mean (SD/IR)	Mean	95% CI	
Education							
Less than high school	9 (3.46)		9 (3.46)				
High school or equivalent	205 (78.8)		205 (78.8)				
College or university	46 (17.7)		46 (17.7)				
Alcohol intake[b]							
Non-drinkers	147 (56.5)		110 (42.3)		-14.2	-20.4, -7.87	0.0047
Light	74 (28.5)		84 (32.3)		3.85	-3.10, 10.7	
Moderate	25 (9.62)		44 (16.9)		7.31	2.31, 12.2	
Heavy	14 (5.38)		22 (8.46)		3.08	-1.03, 7.13	
Current smokers	69 (26.5)		72 (27.7)		1.15	-2.90, 5.17	0.58
Hypertension ≥ stage 1[c]	128 (49.2)		165 (63.5)		14.2	6.65, 21.6	0.0002
Hypertension ≥ stage 2[c]	45 (17.3)		45 (17.3)		--	--	--
Treated hypertension	14 (5.38)		25 (9.62)		4.23	1.55, 6.85	0.0019
Diabetes mellitus[d]	10 (3.85)		16 (6.15)		2.31	0.19, 4.39	0.032

Table S1. Baseline and follow-up characteristics of workers in the DST cohort (continued from page 3). Average values are arithmetic [standard deviation (SD)] or geometric means [interquartile range (IR)]. [CI=confidence interval; HDL=high density lipoprotein; DST=digit-symbol test].

Characteristic (N=260)	Baseline		Last follow-up		△ (95% CI)[a]		P value
	N (%)	Mean (SD/IR)	N (%)	Mean (SD/IR)	Mean	CI	
Age, years		29.4 (9.68)		31.2 (9.75)	1.82	1.76, 1.87	<0.0001
Systolic pressure (mm Hg)[c]		120.1 (10.5)		123.0 (9.67)	2.97	1.66, 4.29	<0.0001
Diastolic pressure (mm Hg)[c]		79.6 (8.93)		80.8 (7.44)	1.22	0.09, 2.36	0.035
Heart rate (beats/minute)[c]		74.3 (11.8)		79.0 (11.1)	4.73	3.05, 6.42	<0.0001
Body mass index (kg/m2)[e]		28.8 (6.04)		29.4 (6.10)	0.62	0.36, 0.89	<0.0001
Serum creatinine (mg/dL)[f]		0.93 (0.23)		1.01 (0.18)	0.08	0.06, 0.10	<0.0001
Total serum cholesterol (mg/dL)[f]		171.8 (38.4)		174.5 (37.3)	2.59	-0.33, 5.50	0.082
HDL serum cholesterol (mg/dL)[f]		46.8 (12.4)		46.2 (11.7)	-0.55	-1.61, 0.51	0.31
Total-to-HDL cholesterol ratio		3.90 (1.30)		4.02 (1.40)	0.12	0.01, 0.22	0.033
Blood glucose (mmol/L)[f]		93.8 (15.6)		91.3 (34.9)	-2.24	-6.79, 2.31	0.33
Blood lead (μg/dL, log)[f]		3.97 (2.30-7.70)		13.1 (9.45-22.1)	230	195, 270	<0.0001

[a] Changes from baseline to last follow-up are given with 95% confidence interval. For proportions, categorical variables and logarithmically transformed variables, percentage changes are given.

[b] Participants were categorized as non-drinker versus light, moderate or heavy drinkers. Among women, light, moderate and heavy drinkers reported a daily alcohol intake of ≤6, >6-14 and >14 gram; for men, these quantities were ≤12, >12-28, and >28 gram, respectively.

[c] Blood pressure was the average of five consecutive readings obtained with the auscultatory technique after the workers had rested for 5 minutes in the seated position. Hypertension was categorized according to the 2017 ACC/AHA guideline, irrespective of treatment status. If systolic and diastolic blood pressures were in different categories, the highest level was used to classify participants. Stage-1 hypertension was a blood pressure of 130-139 mm Hg systolic and 80-89 mm Hg diastolic. Higher levels were classified as stage 2. Heart rate was counted over 15 s.

[d] Diabetes mellitus was a self-reported diagnosis, a fasting blood glucose of 126 mg/dL (7 mmol/L) or higher, or use of antidiabetic drugs.

[e] Body mass index is weight in kilograms divided by height in meter squared.

[f] To convert serum creatinine to μmol/L, multiply by 88.42; to convert cholesterol to mmol/L, multiply by 38.67; to convert glucose to mg/dL, multiply by 17.9; to convert lead from μg/dL to μmol/L multiply by 20.

Table S2. Baseline and follow-up characteristics of workers in the ST cohort (starts). Average values are arithmetic [standard deviation (SD)] or geometric means [interquartile range (IR)]. [CI=confidence interval; HDL=high density lipoprotein; ST=Stroop test].

Characteristic (N=168)	Baseline		Last follow-up		△ (95% CI)[a]		P value
	N (%)	Mean (SD/IR)	N (%)	Mean (SD/IR)	Mean	CI	
Education							
Less than high school	7 (4.17)		7 (4.17)				
High school or equivalent	130 (77.4)		130 (77.4)				
College or university	31 (18.5)		31 (18.5)				
Alcohol intake[b]							
Non-drinkers	95 (56.5)		72 (42.9)		-13.7	-21.7, -5.39	0.067
Light	49 (29.2)		61 (36.3)		7.14	-1.30, 15.5	
Moderate	14 (8.33)		24 (14.3)		5.95	-0.15, 11.9	
Heavy	10 (5.95)		11 (6.55)		0.60	-4.16, 5.34	
Current smokers	47 (28.0)		52 (31.0)		2.98	-2.60, 8.45	0.30
Hypertension ≥ stage 1[c]	80 (47.6)		118 (70.2)		22.6	13.9, 30.8	<0.0001
Hypertension ≥ stage 2[c]	30 (17.9)		36 (21.4)		3.57	-2.30, 9.38	0.24
Treated hypertension	10 (5.95)		14 (8.33)		2.38	-0.45, 5.15	0.10
Diabetes mellitus[d]	6 (3.57)		7 (4.17)		0.60	-1.40, 2.58	0.56

Table S2. Baseline and follow-up characteristics of workers in the ST cohort (continued from page 5). Average values are arithmetic [standard deviation (SD)] or geometric means [interquartile range (IR)]. [CI=confidence interval; HDL=high density lipoprotein; ST=Stroop test].

Characteristic (N=168)	Baseline		Last follow-up		Δ (95% CI)[a]		P value
	N (%)	Mean (SD/IR)	N (%)	Mean (SD/IR)	Mean	95% CI	
Age, years		29.7 (9.68)		30.8 (9.96)	1.10	0.72, 1.49	<0.0001
Systolic pressure (mm Hg)c		119.4 (10.3)		123.4 (9.30)	4.01	2.50, 5.53	<0.0001
Diastolic pressure (mm Hg)c		79.6 (9.10)		82.9 (7.27)	3.27	1.85, 4.69	<0.0001
Heart rate (beats/minute)[c]		74.8 (12.3)		80.0 (12.0)	5.17	3.25, 7.09	<0.0001
Body mass index (kg/m2)[e]		28.6 (5.59)		28.9 (5.55)	0.25	0.03, 0.46	0.026
Serum creatinine (mg/dL)[f]		0.94 (0.17)		1.00 (0.17)	0.06	0.04, 0.08	<0.0001
Total serum cholesterol (mg/dL)[f]		173.7 (34.0)		172.0 (35.9)	-1.68	-5.14, 1.78	0.34
HDL serum cholesterol (mg/dL)[f]		46.5 (12.0)		45.3 (11.7)	-1.25	-2.53, 0.02	0.055
Total-to-HDL cholesterol ratio		3.94 (1.14)		4.03 (1.31)	0.10	-0.03, 0.22	0.12
Blood glucose (mmol/L)[f]		93.8 (16.3)		87.3 (31.9)	-6.48	-11.8, -1.16	0.017
Blood lead (µg/dL, log)[f]		4.13 (2.30-7.80)		14.2 (10.9-21.3)	244	203, 291	<0.0001

[a] Changes from baseline to last follow-up are given with 95% confidence interval. For proportions, categorical variables and logarithmically transformed variables, percentage changes are given.

[b] Participants were categorized as non-drinker versus light, moderate or heavy drinkers. Among women, light, moderate and heavy drinkers reported a daily alcohol intake of ≤6, >6-14 and >14 gram; for men, these quantities were ≤12, >12-28, and >28 gram, respectively.

[c] Blood pressure was the average of five consecutive readings obtained with the auscultatory technique after the workers had rested for 5 minutes in the seated position. Hypertension was categorized according to the 2017 ACC/AHA guideline, irrespective of treatment status. If systolic and diastolic blood pressures were in different categories, the highest level was used to classify participants. Stage 1 hypertension was a blood pressure of 130-139 mm Hg systolic and 80-89 mm Hg diastolic. Higher levels were classified as stage 2. Heart rate was counted over 15 s.

[d] Diabetes mellitus was a self-reported diagnosis, a fasting blood glucose of 126 mg/dL (7 mmol/L) or higher, or use of antidiabetic drugs.

[e] Body mass index is weight in kilograms divided by height in meter squared.

f To convert serum creatinine to µmol/L, multiply by 88.42; to convert cholesterol to mmol/L, multiply by 38.67; to convert glucose to mg/dL, multiply by 17.9; to convert lead from µg/dL to µmol/L multiply by 20.

Table S3. Baseline characteristics of workers analyzed and not analyzed (starts). Average values are arithmetic [standard deviation (SD)] or geometric means [interquartile range (IR)]. [CI=confidence interval; HDL=high density lipoprotein].

Characteristic	Analyzed (N=267)		Not analyzed (N=239)		P value
	N (%)	Mean (SD/IR)	N (%)	Mean (SD/IR)	
Education					
Less than high school	9 (3.4)		6 (2.5)		
High school or equal	211 (79.0)		190 (79.5)		0.61
College or university	47 (17.6)		43 (18.0)		
Alcohol consumption[a]					
Non-drinkers	150 (56.2)		146 (61.1)		
Light	77 (28.8)		63 (26.4)		0.72
Moderate	25 (9.36)		19 (7.95)		
Heavy	15 (5.62)		11 (4.60)		
Current smokers	71 (26.6)		73 (30.5)		0.33
Hypertension > stage 1[b]	132 (49.4)		155 (64.9)		0.0005
Hypertension ≥ stage 2[b]	47 (17.6)		40 (16.7)		0.80
Treated hypertension[b]	14 (5.24)		11 (4.60)		0.74
Diabetes mellitus[c]	10 (3.75)		6 (2.51)		0.43

Table S3. Baseline characteristics of workers analyzed and not analyzed (continued from page 7). Average values are arithmetic [standard deviation (SD)] or geometric means [interquartile range (IR)]. [CI=confidence interval; HDL=high density lipoprotein].

Characteristic	Analyzed (N=267)		Not analyzed (N=239)		P value
	N (%)	Mean (SD/IR)	N (%)	Mean (SD/IR)	
Age, years		29.5 (9.78)		27.9 (10.1)	0.085
Systolic pressure (mm Hg)[b]		120.1 (10.5)		120.2 (10.2)	0.90
Diastolic pressure (mm Hg)[b]		79.6 (8.90)		81.2 (8.24)	0.037
Heart rate (beats/minute)[b]		74.3 (11.9)		74.9 (12.5)	0.59
Body mass index (kg/m2)[d]		28.7 (6.02)		29.2 (6.84)	0.37
Serum creatinine (md/dL)[e]		0.93 (0.23)		0.95 (0.17)	0.83
Total serum cholesterol (mg/dL)[e]		172.0 (38.1)		171.5 (38.4)	0.95
HDL serum cholesterol (mg/dL)[e]		46.9 (12.3)		47.4 (12.0)	0.58
Total-to-HDL cholesterol ratio		3.89 (1.29)		3.85 (1.35)	0.80
Blood glucose (mg/dL)[e]		93.8 (15.4)		93.2 (11.9)	0.33
Blood lead (μg/dL, log)[e]		4.04 (2.30-7.80)		4.14 (2.30-7.80)	0.74

[a] Participants were categorized as non-drinker versus light, moderate and heavy drinkers. Among women, light, moderate and heavy drinkers reported a daily alcohol intake of ≤6, >6-14 and >14 gram; for men, these quantities were ≤12, >12-28, and >28 gram, respectively.

[b] Blood pressure was the average of five consecutive readings obtained with the auscultatory technique after the workers had rested for 5 minutes in the seated position. Hypertension was categorized according to the 2017 ACC/AHA guideline, irrespective of treatment status. If systolic and diastolic blood pressures were in different categories, the highest level was used to classify participants. Stage 1 hypertension was a blood pressure of 130-139 mm Hg systolic and 80-89 mm Hg diastolic. Higher levels were classified as stage 2. Heart rate was counted over 15 s.

[c] Diabetes mellitus was a self-reported diagnosis, a fasting blood glucose of 126 mg/dL (7 mmol/L) or higher, or use of antidiabetic drugs.

[d] Body mass index is weight in kilograms divided by height in meter squared.

[e] To convert serum creatinine to μmol/L, multiply by 88.42; to convert cholesterol to mmol/L, multiply by 38.67; to convert glucose to mg/dL, multiply by 17.9; to convert lead from μg/dL to μmol/L multiply by 20.

Table S4. Baseline neurocognitive function by fourths of the distribution of the follow-up-to-baseline blood lead concentration ratio (starts). Average values are geometric means [interquartile range (IR)]. [MRT=mean reaction time; DST=digit-symbol test; ST=Stroop test].

Characteristic	Low fourth		Low-middle fourth		High-middle fourth		High fourth		P for linear trend[a]
	N/Mean	%/IR	N/Mean	%/IR	N/Mean	%/IR	N/Mean	%/IR	
DST cohort (N=260)									
Quartile limits	<1.90		1.90-3.37		3.37-5.75		≥5.75		
Mean latency time (s, log)	112.8	97.5-130.5	109.8	96.0-120.8	105.9	97.6-116.1	107.2	92.8-119.1	0.076
Number of errors									
0	33	50.8	37	56.9	43	66.2	40	61.5	
1	21	32.3	17	26.2	18	27.7	17	26.2	0.10
>1	11	16.9	11	16.9	4	6.2	8	12.3	
ST cohort (N=168)									
Quartile limits	<1.98		1.98-3.26		3.26-5.52		≥5.52		
MRT in incongruent trials (ms, log)									
All responses	1636	1321-1965	1552	1238-1841	1574	1348-1837	1664	1356-2006	0.75
Correct responses[a]	1638	1321-1965	1559	1238-1876	1572	1373-1837	1666	1356-2047	0.78
MRT congruent trials (ms, log)									
All responses	1495	1187-1815	1390	1163-1492	1565	1195-1920	1496	1179-1680	0.61
Correct responses[a]	1495	1187-1815	1390	1163-1492	1565	1195-1920	1496	1179-1680	0.61

Table S4. Baseline neurocognitive function by fourths of the follow-up-to-baseline blood lead concentration ratio (continued from page 9). Average values are geometric means [interquartile range (IR)]. [MRT=mean reaction time; DST=digit-symbol test; ST=Stroop test].

Characteristic	Low fourth		Low-middle fourth		High-middle fourth		High quartile		P for linear trend[a]
	N/Mean	%/IR	N/Mean	%/IR	N/Mean	%/IR	N/Mean	%/IR	
Correct ratio in incongruent trials (%)									
100	37	88.1	37	88.1	34	81.0	37	88.1	
90-99	3	7.1	2	4.8	4	9.5	3	7.1	0.40
<90	2	4.8	3	7.1	4	9.5	2	4.8	
Correct ratio in congruent trials (%)									
100	42	100	42	100	42	100	42	100	1.00
<100	0	0	0	0	0	0	0	0	
Interference effect (ms, log)									
All responses	1.09	0.96-1.21	1.12	0.97-1.27	1.01	0.94-1.15	1.11	0.99-1.29	0.77
Correct responses[b]	1.10	0.97-1.22	1.12	0.98-1.27	1.00	0.94-1.15	1.12	0.99-1.19	
Interference score									
>0	0	0.0	0	0.0	0	0.0	0	0.0	
0	37	88.1	37	88.1	34	81.0	37	88.1	0.76
>0	5	11.9	5	11.9	8	19.0	5	11.9	

[a] There was no bias in the initial values of the neurocognitive tests across increasing categories of the follow-up-to-baseline blood lead concentration ratio.
[b] One participant did not provide any correct response at baseline and follow-up and was not included in the MRT of correct responses.

Table S5 Follow-up neurocognitive function by fourths of the distribution of the follow-up-to-baseline blood lead concentration ratio (starts). Average values are geometric means [interquartile range (IR)]. [MRT=mean reaction time; DST=digit-symbol test; ST=Stroop test].

Characteristic	Low fourth		Low-middle fourth		High-middle fourth		High fourth		P for linear trend[a]
	N/Mean	%/IR	N/Mean	%/IR	N/Mean	%/IR	N/Mean	%/IR	
DST cohort (N=260)									
Quartile limits	<1.90		1.90-3.37		3.37-5.75		≥5.75		
Mean latency time (s, log)	104.8	90.8-117.9	111.6	95.5-127.7	105.6	88.7–121.2	108.6	91.3-126.8	0.65
Number of errors									
0	38	58.5	40	61.5	39	60.0	43	66.2	
1	19	29.2	18	27.7	16	24.6	18	27.7	0.38
>1	8	12.3	7	10.8	10	15.4	4	6.2	
ST cohort (N=168)									
Quartile limits	<1.98		1.98-3.26		3.26-5.52		≥5.52		
MRT in incongruent trials (ms, log)									
All responses	2287	1910-2901	2094	1664-2575	2105	1667-2630	1886	1558-2227	0.016
Correct responses[a]	2260	1910-2895	2110	1733-2575	2127	1667-2630	1832	1517-2146	0.0093
MRT congruent trials (ms, log)									
All responses	2237	1854-2763	1883	1501-2535	1935	1539-2248	1882	1539-2099	0.050
Correct responses[a]	2237	1854-2763	1903	1501-2604	1935	1539-2248	1904	1603-2099	0.061

Table S5 Follow-up neurocognitive function by fourths of the distribution of the blood lead changes (continued from page 11). Average values are geometric means [interquartile range (IR)]. [MRT=mean reaction time; DST=digit-symbol test; ST=Stroop test].

Characteristic	Low fourth		Low-middle fourth		High-middle fourth		High fourth		P for linear trend[a]
	N/Mean	%/IR	N/Mean	%/IR	N/Mean	%/IR	N/Mean	%/IR	
Correct ratio in incongruent trials (%)									
100	34	81.0	33	78.6	34	81.0	37	88.1	
90-99	6	14.3	5	11.9	4	9.5	2	4.8	0.10
<90	2	4.8	4	9.5	4	9.5	3	7.1	
Correct ratio in congruent trials (%)									
100	42	100	40	95.2	42	100	40	95.2	0.44
<100	0	0	2	4.8	0	0	2	4.8	
Interference effect (ms, log)									
All responses	1.02	0.88-1.17	1.11	0.93-1.22	1.09	0.93-1.27	1.00	0.85-1.23	0.63
Correct responses[b]	1.02	0.89-1.13	1.10	0.90-1.19	1.10	0.93-1.34	0.98	0.84-1.15	0.52
Interference score									
>0	0	0.0	2	4.8	0	0.0	1	2.4	
0	34	81.0	32	76.2	34	81.0	36	85.7	0.40
>0	8	19.0	8	19.0	8	19.0	5	11.9	

[a] There was no gradient at follow-up in the neurocognitive tests results across increasing categories of the follow-up-to-baseline blood lead concentration ratio.
[b] One participant did not provide any correct response at baseline and follow-up and was not included in the MRT of correct responses.

Table S6. Associations between changes (Δ) from baseline to follow-up in neurocognitive function and blood lead stratified by the median baseline age. [OR=odds ratio; CI=95% confidence interval; DST=digit-symbol test; ST=Stroop test; MRT=mean reaction time].

Characteristic	Stratified by the median baseline age								P for interaction
	%[a]	OR[a]	CI	P value	%[a]	OR[a]	CI	P value	
DST cohort (N=260)									
Age	<26.4 years (N=130)				≥26.4 years (N=130)				
Δ latency time (%)	0.33		-1.04, 1.71	0.64	0.58		-0.65, 1.82	0.35	0.20
Increasing error rate (0,1)		1.14	0.69, 1.89	0.60		1.46	0.89, 2.62	0.13	0.59
ST cohort (N=168)									
Age	<27.0 years (N=84)				≥27.0 years (N=84)				
Δ MRT in incongruent trials									
All responses (%)	1.90		-2.29, 6.25	0.35	-2.58		-5.71, -0.66	0.11	0.57
Correct responses (%)	0.68		-3.24, 4.76	0.72	-2.52		-5.79, 0.86	0.13	0.98
Δ MRT in congruent trials									
All responses (%)	-0.19		-4.72, 4.56	0.93	-1.88		-5.82, 2.23	0.34	0.92
Correct responses (%)	-0.13		-4.71, 4.68	0.95	-1.98		-5.84, 2.04	0.31	0.83
Increasing error rate									
Incongruent trials (0,1)[b]		…	…	…		…	…	…	…
Congruent trials (0,1)[b]		…	…	…		…	…	…	…
Δ Interference effect									
All responses (%)	2.33		-1.03, 5.80	0.16	-0.53		-3.54, 2.56	0.72	0.42
Correct responses (%)	1.91		-1.44, 5.38	0.25	-0.23		-3.41, 3.06	0.88	0.56

[a] For continuous outcomes, estimates express the percentage difference in the follow-up to baseline ratio of DST latency/ST mean reaction time associated with a doubling in the follow-up-to-baseline blood lead concentration ratio. For categorical outcomes, estimates are odds ratios (OR) associating the probability that workers had increasing error rates with a doubling in the follow-up-to-baseline blood lead concentration ratio. Estimates were derived from mixed models, including both the 1-year and 2-year changes in neurocognitive function and blood lead, and accounting for the within-subject correlations using a random participant effect. Adjustments were made for sex, baseline age, the baseline neurocognitive test result, ethnicity (white vs other), change in age, baseline body mass index, change in body weight, educational attainment, baseline blood lead, and the baseline values of and changes during follow-up in smoking status, alcohol consumption (light, moderate and heavy drinkers), and the total-to-HDL serum cholesterol ratio.

[b] An ellipsis indicates that the model did not converge.

Table S7. Associations between changes (Δ) from baseline to follow-up in neurocognitive function and blood lead stratified by the median baseline blood lead concentration [OR=odds ratio; CI=95% confidence interval; DST=digit-symbol test; ST=Stroop test; MRT=mean reaction time].

Characteristic	Stratified by the median baseline blood lead								P for interaction
	%[a]	OR[a]	CI	P value	%[a]	OR[a]	CI	P value	
DST cohort (N=260)									
Baseline blood lead	<4.20 µg/dL (N=126)				≥4.20 µg/dL (N=134)				
Δ latency time (%)	-0.09		-1.22, 1.06	0.88	1.19		-0.29, 2.68	0.11	0.27
Increasing error rate (0,1)		1.00	0.64, 1.58	0.99		1.68	0.99, 2.86	0.056	0.34
ST cohort (N=168)									
Baseline blood lead	<4.30 µg/dL (N=81)				≥4.30 µg/dL (N=87)				
Δ MRT in incongruent trials									
All responses (%)	-1.90		-5.52, 1.86	0.29	1.16		-3.04, 5.53	0.57	0.70
Correct responses (%)	-2.84		-6.16, 0.60	0.096	1.34		-3.08, 5.96	0.54	0.45
Δ MRT in congruent trials									
All responses (%)	-1.70		-5.66, 2.41	0.38	0.12		-5.20, 5.73	0.96	0.65
Correct responses (%)	-1.66		-5.54, 2.39	0.38	0.36		-5.03, 6.05	0.89	0.64
Increasing error rate									
Incongruent trials (0,1)[b]		…	…	…		…	…	…	…
Congruent trials (0,1)[b]		…	…	…		…	…	…	…
Δ Interference effect									
All responses (%)	0.18		-2.47, 2.91	0.88	1.44		-2.67, 5.73	0.47	0.34
Correct responses (%)	-0.18		-2.78, 2.49	0.89	1.55		-2.72, 6.01	0.46	0.22

[a] For continuous outcomes, estimates express the percentage difference in the follow-up to baseline ratio of DST latency/ST mean reaction time associated with a doubling in the follow-up-to-baseline blood lead concentration ratio. For categorical outcomes, estimates are odds ratios (OR) associating the probability that workers had increasing error rates with a doubling in the follow-up-to-baseline blood lead concentration ratio. Estimates were derived from mixed models, including both the 1-year and 2-year changes in neurocognitive function and blood lead, and accounting for within-subject correlations using a random participant effect. Adjustments were made for sex, baseline age, the baseline neurocognitive test result, ethnicity (white vs other), change in age, baseline body mass index, changes in body weight, educational attainment, baseline blood lead, and the baseline values of and changes during follow-up in smoking status, alcohol consumption (light, moderate and heavy drinkers), and the total-to-HDL serum cholesterol ratio.

[b] An ellipsis indicates that the model did not converge.

Table S8. Associations between changes (Δ) from baseline to follow-up in neurocognitive function and blood lead stratified by the median CBLI [OR=odds ratio; CI=95% confidence interval; DST=digit-symbol test; ST=Stroop test; MRT=mean reaction time; CBLI=cumulative blood lead index].

Characteristic	Stratified by the median of CBLI								P for interaction
	%[a]	OR[a]	CI	P value	%[a]	OR[a]	CI	P value	
DST cohort (N=260)									
CBLI	<32.5 μg/dL × year (N=130)				≥32.5 μg/dL × year (N=130)				
Δ latency time (%)	0.57		-0.56, 1.72	0.32	-0.06		-1.53, 1.43	0.93	0.21
Increasing error rate (0,1)		1.10	0.66, 1.83	0.72		1.23	0.75, 2.00	0.41	0.87
ST cohort (N=168)									
CBLI	<33.3 μg/dL × year (N=84)				≥33.3 μg/dL × year (N=84)				
Δ MRT in incongruent trials									
All responses (%)	-0.27		-3.86, 3.46	0.88	-3.12		-6.92, 0.84	0.11	0.57
Correct responses (%)	-1.07		-4.38, 2.35	0.51	-2.80		-6.70, 1.27	0.16	0.83
Δ MRT in congruent trials									
All responses (%)	-0.38		-4.50, 3.91	0.85	-3.80		-8.43, 1.06	0.11	0.69
Correct responses (%)	-0.28		-4.34, 3.96	0.89	-3.75		-8.43, 1.16	0.12	0.64
Increasing error rate									
Incongruent trials (0,1)[b]		…	…	…		…	…	…	…
Congruent trials (0,1)[b]		…	…	…		…	…	…	…
Δ Interference effect									
All responses (%)	0.06		-2.71, 2.91	0.96	1.22		-2.66, 5.24	0.52	0.93
Correct responses (%)	-0.24		-2.91, 2.51	0.85	1.81		-2.20, 5.99	0.36	0.73

[a] For continuous outcomes, estimates express the percentage difference in the follow-up to baseline ratio of DST latency/ST mean reaction time associated with a doubling in the follow-up-to-baseline blood lead ratio. For categorical outcomes, estimates are odds ratios (OR) associating the probability that workers had increasing error rates with a doubling in the follow-up-to-baseline blood lead ratio. Estimates were derived from mixed models, including both the 1-year and 2-year changes in neurocognitive function and blood lead, and accounting for within-subject correlations using a random participant effect. Adjustments were made for sex, baseline age, the baseline neurocognitive test result, ethnicity (white vs other), change in age, baseline body mass index, change in body weight, educational attainment, baseline blood lead, and the baseline values of and changes during follow-up in smoking status, alcohol consumption (light, moderate and heavy drinkers), and the total-to-HDL serum cholesterol ratio.

[b] An ellipsis indicates that the model did not converge.

Table S9. Associations between changes in neurocognitive function (Δ) and blood lead from baseline to follow-up modelled by linear regression. [CI=95% confidence interval; DST=digit-symbol test; ST=Stroop test; MRT=mean reaction time].

Characteristic	Unadjusted			Adjusted[a]			Fully adjusted[b]		
	% [c]	CI	P value	% [c]	CI	P value	% [c]	CI	P value
DST cohort									
1-year follow-up (N=244)									
Δ latency time (%)	0.49	-0.30, 1.29	0.23	0.13	-0.53, 0.80	0.69	-0.08	-1.06, 0.90	0.87
2-year follow-up (N=203)									
Δ latency time (%)	1.72	0.62, 2.84	0.0025	0.97	0.03, 1.92	0.044	1.37	0.05, 2.71	0.043
ST cohort [d]									
1-year follow-up (N=168)									
Δ MRT in incongruent trials	-2.15	-3.95, -0.31	0.023	-1.96	-3.48, -0.42	0.014	-1.07	-3.47, 1.38	0.39
Δ MRT in congruent trials	-1.24	-3.47, 1.03	0.28	-1.19	-2.94, 0.58	0.19	-1.93	-4.66, 0.88	0.18
Δ Interference effect	-0.92	-2.67, 0.87	0.31	-0.72	-2.08, 0.67	0.31	1.31	-0.88, 3.55	0.24

[a] Adjusted models accounted for sex and baseline age and the baseline neurocognitive test results, i.e., latency/reaction time (continuous outcomes) or the number of errors (ordinal outcomes).

[b] Fully adjusted models additionally accounted for ethnicity (white *vs* other), change in age, baseline body mass index, change in body weight, educational attainment, baseline blood lead, and the baseline values of and changes during follow-up in smoking status, the total-to-HDL serum cholesterol ratio, and alcohol intake (light, moderate and heavy).

[c] All association sizes are expressed for a doubling in the follow-up-to-baseline blood lead concentration ratio. Estimates are the percentage difference in the follow-up minus baseline value for continuous variables and odds ratios for categorical outcomes.

[d] Only 48 participants had a follow-up visit at the second year.

[e] An ellipsis indicates that the model did not converge.

Table S10. Association of neurocognitive function with blood lead at baseline and last follow-up analysed separately, using linear of logistic regression. [CI=95% confidence interval; MRT=mean reaction time; BL=baseline; FU=last follow-up; P*slope*=significance of the difference between baseline and follow-up in the slope of the neurocognitive measurement on the blood lead concentration]

Characteristics		Unadjusted[a]				Adjusted[a]			
Stroop test		β	95% CI	P-value	P*slope*	β	95% CI	P-Value	P*slope*
MRT of incongruent trials (ms, log)	BL	1.53	-4.29, 7.71	0.61	0.92	1.66	-4.27, 7.95	0.59	0.57
	FU	1.00	-7.56, 10.4	0.83		4.97	-4.30, 15.1	0.31	
MRT of congruent trials (ms, log)	BL	0.80	-5.79, 7.84	0.82	0.67	0.71	-6.02, 7.92	0.84	0.88
	FU	-1.70	-10.5, 7.92	0.72		1.67	-8.16, 12.6	0.75	
Interference effect	BL	0.73	-3.64, 5.30	0.75	0.61	0.94	-3.53, 5.62	0.69	0.59
	FU	2.75	-3.50, 9.40	0.40		3.25	-3.58, 10.6	0.36	
Digit-symbol test		β	95% CI	P-value	P*slope*	β	95% CI	P-Value	P*slope*
Latency time (s, log)	BL	0.38	-2.47, 3.31	0.80	0.47	1.04	-1.82, 3.98	0.48	0.75
	FU	2.10	-1.47, 5.81	0.25		1.85	-2.03, 5.88	0.36	
Stroop Test		OR	95% CI	P-Value	P*slope*	OR	95% CI	P-Value	P*slope*
Error score in incongruent trials	BL	1.53	0.41, 5.69	0.53	0.34	1.35	0.32, 0.55	0.68	0.32
	FU	0.60	0.15, 2.45	0.48		0.46	0.10, 2.16	0.33	
Interference score	BL	0.65	0.18, 2.43	0.53	0.28	0.74	0.18, 3.07	0.68	0.28
	FU	1.88	0.46, 7.71	0.38		2.36	0.50, 11.2	0.28	
Digit-symbol test		OR	95% CI	P-Value	P*slope*	OR	95% CI	P-Value	P*slope*
Error score	BL	0.93	0.47, 1.81	0.82	0.33	0.97	0.48, 1.98	0.94	0.23
	FU	1.56	0.70, 3.48	0.28		2.00	0.80, 5.00	0.14	

[a] All models were derived by linear or logistic regression analysis for continuous and categorical outcomes, respectively. Adjusted models accounted for sex, age, ethnicity (white vs other), body mass index, educational attainment, current smoking, alcohol intake (light, moderate and heavy), and the total-to-HDL serum cholesterol ratio. Associatiion sizes were expressed for a 10-fold difference in the blood lead concentration at baseline or follow-up.

Table S11. Time-dependent DST and ST results by observer (starts). Average values are geometric means [interquartile range (IR)]. [MRT=mean reaction time; DST=digit-symbol test; ST=Stroop test].

Characteristic[a]	Overall			Baseline			Year 1			Year 2		
	N[b]	Mean	IR	N[b]	Mean	IR	N[b]	Mean	IR	N[b]	Mean	IR
DST cohort (N=260)												
Mean latency time (s, log)[c]												
Observer 1	6	99.1	84.4-111.8	6	99.1	84.4-111.8	0	…	…	0	…	…
Observer 2	57	101.1	89.9-114.4	53	101.8	91.6-114.4	2	106.3	89.2-126.8	2	79.6	64.2-98.6
Observer 3	72	104.1	93.6-117.4	69	104.6	94.4-117.3	3	93.3	77.8-118.0	0	…	…
Observer 4	99	108.0	91.8-122.3	0	…	…	39	115.5	102.1-130.0	60	103.4	88.1-115.8
Observer 5	118	111.5	93.0-131.9	0	…	…	40	119.3	107.0-133.6	78	107.6	90.0-125.9
Observer 6	40	112.0	97.6-124.8	28	109.5	95.1-121.6	12	118.0	108.3-131.8	0	…	…
Observer 7	75	112.1	100.8-122.7	41	107.7	95.6-119.9	34	117.6	108.6-123.4	0	…	…
Observer 8	7	112.6	87.7-131.3	3	120.6	85.3-156.5	4	107.0	93.9-121.9	0	…	…
Observer 9	148	115.7	102.7-130.2	37	120.6	107.4-131.2	70	117.7	105.7-131.5	41	108.3	91.3-120.3
Observer 10	85	116.9	101.8-133.5	23	124.5	104.8-149.8	40	119.3	107.0-133.6	22	105.4	91.3-125.8
ST cohort (N=168)												
MRT in incongruent trials (ms, log)[c]												
Observer 1	5	1344	1294-1499	5	1344	1294-1499	0	…	…	0	…	…
Observer 2	5	1426	1146-1440	1	1225	…	4	1481	1129-1942	0	…	…
Observer 3	52	1471	1216-1734	49	1468	1235-1725	3	1515	1089-2450	0	…	…
Observer 4	39	1659	1288-1998	37	1678	1307-1998	2	1346	1051-1724	0	…	…
Observer 5	31	1693	1321-2081	21	1466	1210-1842	10	2291	2014-2533	0	…	…
Observer 6	78	1714	1410-2014	40	1695	1371-1946	38	1734	1425-2150	0	…	…
Observer 7	32	1926	1608-2250	0	…	…	28	1855	1516-2116	4	2504	2069-3030
Observer 8	87	2100	1667-2535	15	2049	1513-2678	63	2104	1672-2516	9	2155	1736-2630
Observer 9	17	2154	1919-2478	0	…	…	8	2012	1605-2420	9	2288	1919-2758
Observer 10	32	2521	1942-3311	0	…	…	12	2317	1891-3176	20	2652	2038-3314

Table S11. Time-dependent DST and ST results by observer (continued from page 18). Average values are geometric means [interquartile range (IR)]. [MRT=mean reaction time; DST=digit-symbol test; ST=Stroop test].

Characteristic[a]	Overall			Baseline			Year 1			Year 2		
	N[b]	Mean	IR	N[b]	Mean	IR	N[b]	Mean	IR	N[b]	Mean	IR
MRT in congruent trials (ms, log)[c]												
Observer 1	5	1238	1125-1428	5	1238	1125-1428	0	…	…	0	…	…
Observer 2	5	1262	1103-1255	1	1255	…	4	1264	1084-1473	0	…	…
Observer 3	52	1343	1110-1567	49	1342	1119-1585	3	1362	1046-2084	0	…	…
Observer 4	31	1477	1173-1999	21	1280	1106-1467	10	1997	1894-2239	0	…	…
Observer 5	78	1610	1298-1859	40	1542	1276-1830	38	1685	1390—2078	0	…	…
Observer 6	39	1620	1246-1920	37	1644	1273-1920	2	1232	1062-1430	0	…	…
Observer 7	32	1824	1461-2198	0	…	…	28	1814	1461-2198	4	1898	1639-2198
Observer 8	87	1961	1570-2375	15	1925	1335-2327	63	1961	1598-2375	9	2026	1682-2844
Observer 9	17	2173	1556-2827	0	…	…	8	1907	1429-2384	9	2440	2057-2853
Observer 10	32	2399	1680-3134	0	…	…	12	2138	1646-2639	20	2571	1939-3189

[a] Observers were sorted according to the participants' performance in all neurocognitive test they took from the workers. Braces join study nurses with similar mean latency time or MRT among the workers undergoing testing.

[b] N indicates the number of tests.

[c] Significance of the difference between observers derived by ANOVA: $P<0.0001$.

Table S12. Geometric mean blood lead levels in the DST and ST cohorts by observer. [interquartile range (IR)]. [DST=digit-symbol test; ST=Stroop test].

Characteristic[a]	Overall			Baseline			Year 1			Year 2		
	N[b]	Mean	IR	N[b]	Mean	IR	N[b]	Mean	IR	N[b]	Mean	IR
Blood lead in DST cohort (μg/dL, N=260)[c]												
Observer 1	6	1.4	0.6-2.2	6	1.4	0.6-2.2	0	…	…	0	…	…
Observer 2	72	4.2	2.6-7.2	69	3.9	2.6-6.5	3	13.6	7.1-24.5	0	…	…
Observer 3	57	4.3	2.8-7.7	53	4.0	2.5-7.3	2	14.5	12.1-17.4	2	10.9	5.0-23.7
Observer 4	75	7.7	4.2-18.0	41	4.0	1.8-8.3	34	17.2	12.3-22.2	0	…	…
Observer 5	40	8.1	3.0-17.9	28	5.9	2.5-11.9	12	17.1	14.6-22.8	0	…	…
Observer 6	7	8.2	4.0-15.9	3	4.6	3.0-8.3	4	12.7	7.7-21.0	0	…	…
Observer 7	85	9.0	4.9-18.7	23	3.4	1.6-9.0	40	12.6	8.6-22.2	22	13.3	10.3-17.6
Observer 8	148	9.6	5.2-18.6	37	3.8	2.0-7.2	70	13.1	8.6-20.6	41	13.0	11.7-19.4
Observer 9	118	12.1	8.5-22.1	0	…	…	40	13.2	10.6-22.6	78	11.6	7.4-21.8
Observer 10	99	13.0	8.8-21.1	0	…	…	39	11.3	7.8-19.5	60	14.2	11.1-21.9
Blood lead in ST cohort (μg/dL, N=168)[c]												
Observer 1	5	1.5	0.6-2.2	5	1.5	0.6-2.2	0	…	…	0	…	…
Observer 2	52	3.9	2.6-6.4	49	3.6	2.6-5.7	3	13.6	7.1-24.5	0	…	…
Observer 3	39	4.4	2.8-8.3	37	4.1	2.8-7.3	2	14.5	12.1-17.4	0	…	…
Observer 4	31	8.4	3.7-15.9	21	6.0	2.9-11.8	10	16.7	14.7-23.1	0	…	…
Observer 5	78	8.4	4.3-19.1	40	4.2	1.8-8.7	38	17.7	12.9-23.9	0	…	…
Observer 6	87	11.1	6.8-19.2	15	5.0	3.2-10.0	63	13.4	8.3-20.6	9	11.7	11.0-14.8
Observer 7	5	11.7	8.3-15.9	1	8.3	…	4	12.7	7.7-21.0	0	…	…
Observer 8	32	12.2	9.4-19.9	0	…	…	28	12.2	9.4-19.9	4	11.9	7.7-18.3
Observer 9	17	16.6	14.1-20.4	0	…	…	8	14.1	12.4-16.5	9	19.1	15.9-24.6
Observer 10	32	17.3	14.8-24.5	0	…	…	12	15.6	15.1-23.8	20	18.3	14.8-25.3

[a] Observers were sorted according to the participants' baseline blood lead concentration. Braces join study nurses with similar blood lead levels in tested workers.
[b] N indicates the number of tests.
[c] Significance of the difference between observers derived by ANOVA: $P<0.0001$.

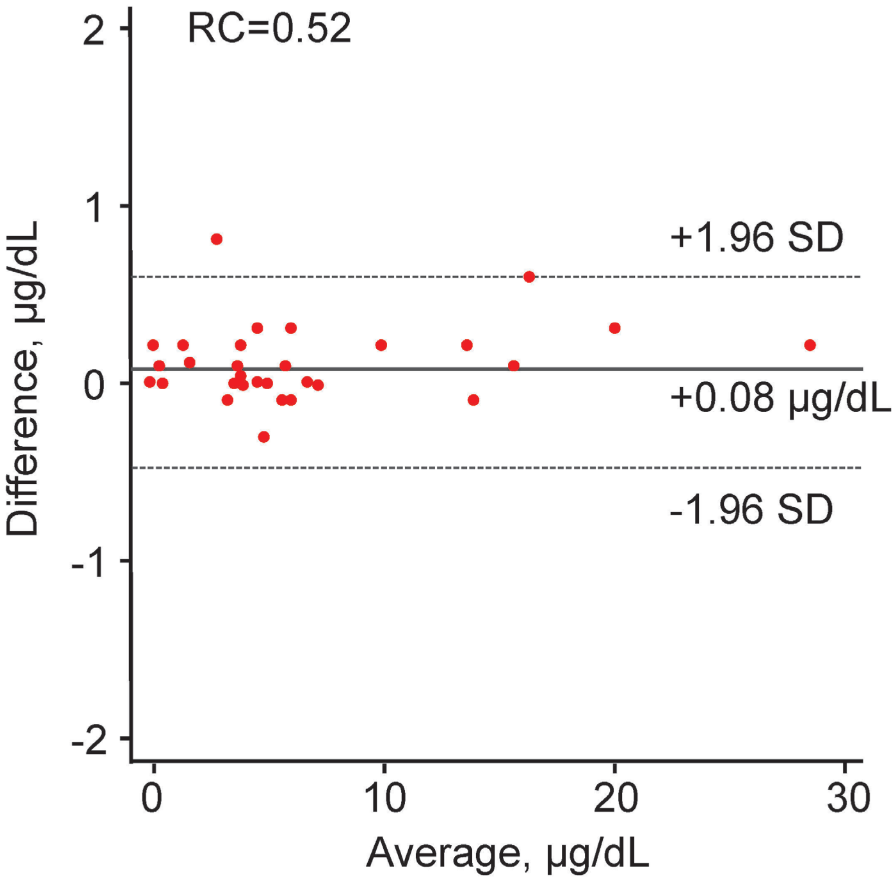

Figure S1

Bland–Altman plot for duplicate blood lead measurements in 30 workers. The difference between both measurements was plotted against the average of both measurements. The bias (repeat minus first measurement) was +0.08 µg/dL (P=0.078). The reproducibility coefficient (RC) is twice the SD of the signed differences between duplicate measurements. Reproduced from reference 17.

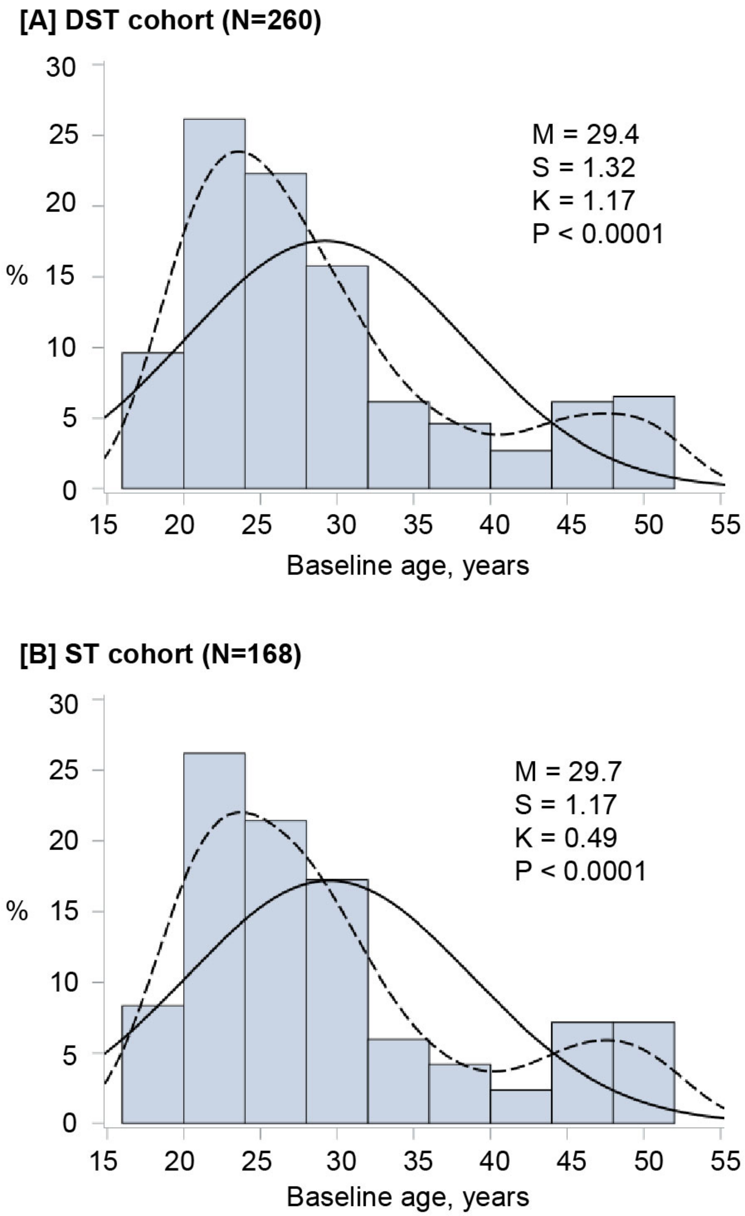

Figure S2

Baseline age distribution in the DST [A] and ST [B] cohorts. [M, mean; S, skewness; K, kurtosis; DST=digit-symbol test; ST=Stroop test]. The solid and dotted lines represent the normal and kernel density distributions. The P values are for departure of the actually observed distribution from normality according to the Shapiro-Wilk statistic.

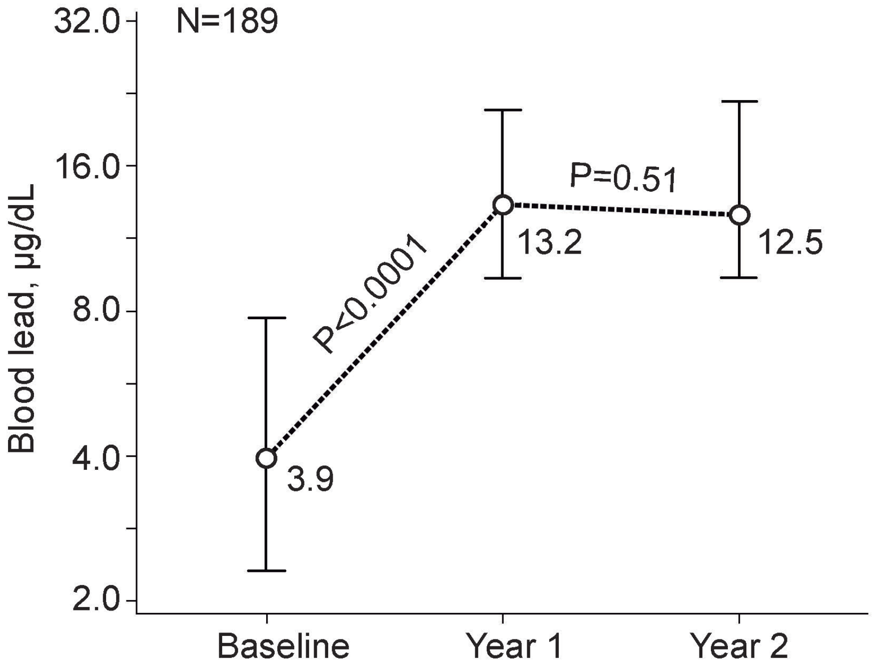

Figure S3

Blood lead levels at baseline and at the 1-year and 2-year follow-up visits in 189 workers with 2 follow-up visits. Plotted values are geometric means. Vertical bars indicate the interquatile range.

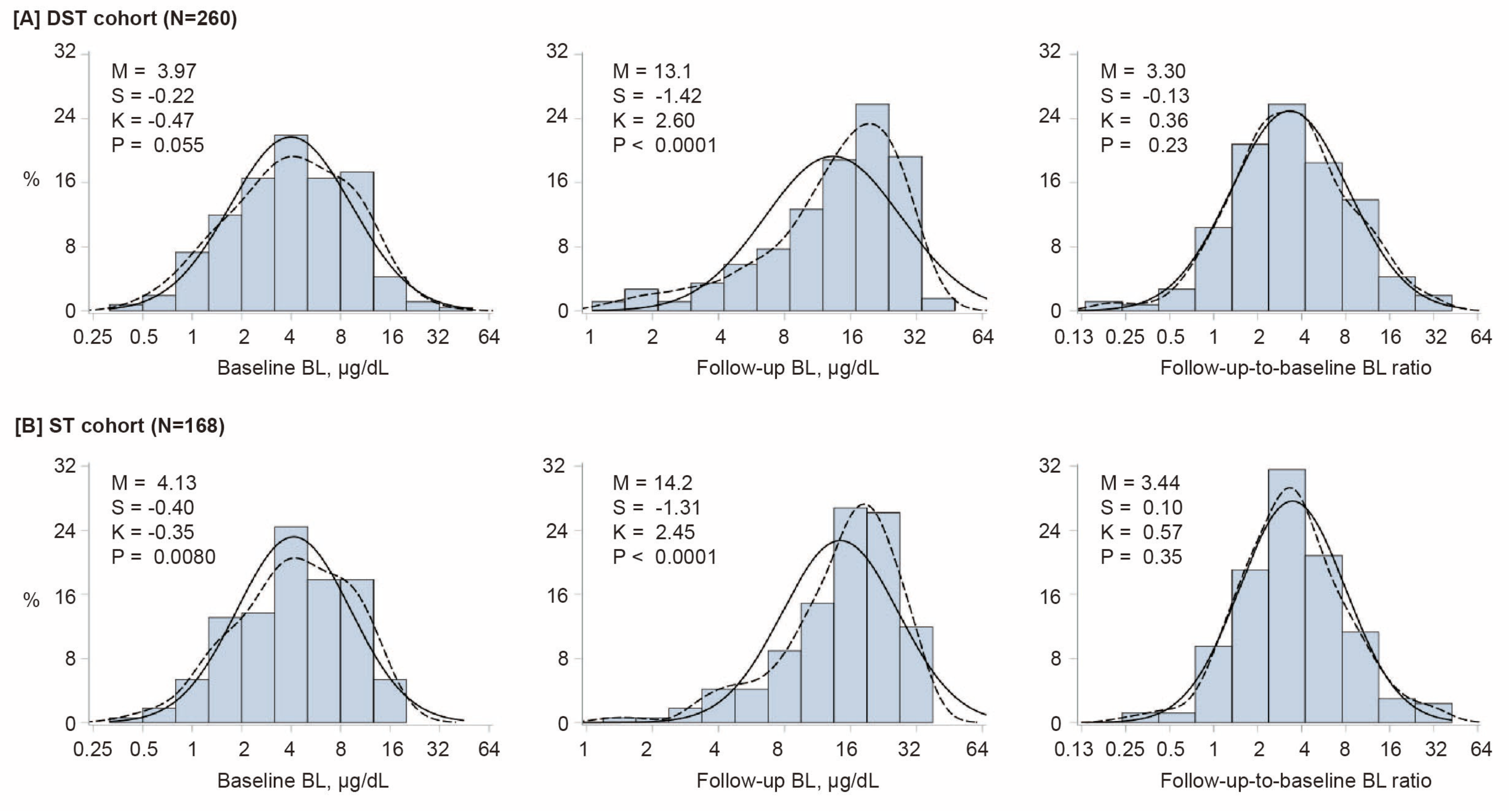

Figure S4

Distributions of baseline blood lead, follow-up blood lead, and the baseline-to-last-follow-up ratios in blood lead in the DST and ST cohorts. [M, mean; S, skewness; K, kurtosis; DST=digit-symbol test; ST=Stroop test; BL=blood lead]. The solid and dotted lines represent the normal and kernel density distributions. The P values are for departure of the actually observed distribution from normality according to the Shapiro-Wilk statistic.

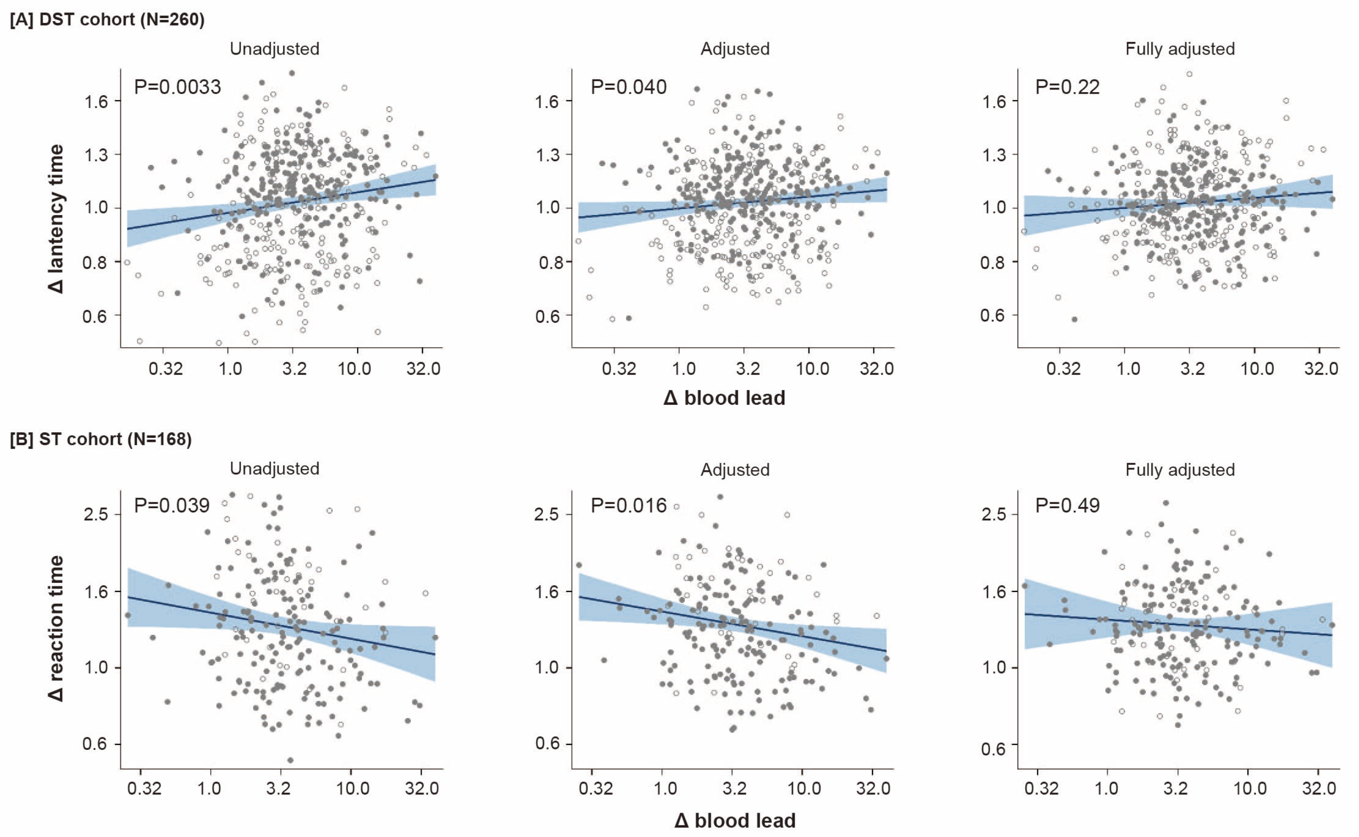

Figure S5

Associations of the changes from baseline to last follow-up (Δ) in latency time in DST cohort and in mean reaction time in ST cohort with the follow-up-to-baseline blood lead concentration ratio (Δ). [DST=digit-symbol test; ST=Stroop test]. Closed and open symbols depict the first and second follow-up results, respectively. The regression line with 95% confidence interval were derived from mixed models accounting for clustering of the observations within participants. Adjusted models accounted for sex, baseline age and the baseline value of latency time or mean reaction time. Fully adjusted models additionally accounted for for ethnicity (white vs other), change in age, baseline body mass index, changes in body weight, educational attainment, baseline blood lead, and the baseline values of and changes during follow-up in smoking status, and the total-to-HDL serum cholesterol ratio and alcohol consumption (light, moderate and heavy drinkers).
